# Unmasking immunodeficiency in sudden infant deaths: no evidence of hidden immune disorders

**DOI:** 10.3389/fimmu.2026.1860011

**Published:** 2026-08-05

**Authors:** Jintana Bunpan Andersen, Janne Maren Strand, Asbjørg Stray-Pedersen, Tore Gunnar Abrahamsen, Arne Stray-Pedersen, Trond Flægstad, Anders Moen, Franziskus Johannes Bosse, Hans Christian Erichsen Landsverk

**Affiliations:** 1 Department of Medicine, Sørlandet Hospital, Kristiansand, Norway; 2 National Center for Rare Disorders and Department of Pediatric Research, Oslo University Hospital, Oslo, Norway; 3 Norwegian National Unit for Newborn Screening, Oslo University Hospital, Oslo, Norway; 4 Institute of Clinical Medicine, University of Oslo, Oslo, Norway; 5 Department of Forensic Sciences, Oslo University Hospital, Oslo, Norway; 6 Department of Forensic Medicine, University of Oslo, Oslo, Norway; 7 Department of Pediatrics, University Hospital of North Norway, Tromsø, Norway; 8 Department of Pediatrics, Ålesund Hospital, Ålesund, Norway; 9 Department of Pediatric and Adolescent Medicine, Haukeland University Hospital, Bergen, Norway; 10 Department of Pediatrics, Oslo University Hospital, Oslo, Norway

**Keywords:** cause of death registry, inborn errors of immunity, infant mortality, newborn screening, population based study, primary immunodeficiency, severe combined immunodeficiency, sudden infant death

## Abstract

**Introduction:**

Sudden infant death syndrome (SIDS) remains a leading cause of post-neonatal mortality, yet its underlying mechanisms are largely unknown. Evidence suggests that immune dysfunction may play a role in a subset of cases. However, the role of inborn errors of immunity (IEI), particularly severe combined immunodeficiency (SCID), has not been systematically explored. We aimed to study their potential role in SIDS and infant deaths from infectious disease.

**Methods:**

We obtained mortality data for 2012–2017 from The Norwegian Cause of Death Registry and identified eligible infants by reviewing ICD-10 codes for infection-related deaths or SIDS. The medical history and autopsy reports were reviewed and infants with predisposition to infection were excluded. Genetic testing was performed on biobanked newborn dried blood spot samples following the protocol for identifying SCID/IEI in live-born infants.

**Results:**

The study population consisted of 79 infants, of which 66% (*n* = 52) died of SIDS and 34% (*n* = 27) due to infection. The majority of the infants in both groups had at least one genetic IEI-related variant, but no findings were clearly pathogenic. Variants of uncertain significance in autosomal dominant disease genes were identified in 18% (*n* = 14). Heterozygous pathogenic variants in autosomal recessive genes were identified in 23% (*n* = 18). Risk factor variants were identified in 42% (*n* = 33), including two mannose-binding lectin 2 polymorphisms with alleles more frequently observed in the infection group compared to the general population.

**Discussion:**

No missed cases of SCID/IEI were identified among infants who died from infections or SIDS. Yet a significant proportion of the cohort may carry genetic predispositions due to the presence of immune-related genetic variants. Even in the absence of overt immunodeficiency, these findings may reflect a latent vulnerability interacting with physiological and environmental factors.

## 1 Introduction

Sudden infant death syndrome (SIDS) and infections remain significant contributors to post-neonatal mortality in the developed world (1). The pathogenesis of SIDS is poorly understood, and the identification of potential underlying causes has been challenging. By definition, the cause of death in SIDS remains unexplained despite a thorough autopsy including histological assessment and microbiological analyses (2). Nevertheless, both epidemiological and experimental laboratory data indicate that inflammation and infections might play a role in a subset of SIDS victims (2–5). In half of SIDS cases, a mild respiratory infection was reported at the last days prior to their death (5). Elevated pro-inflammatory cytokine levels, activated mucosal immune cells and altered gut microbiome have been reported in a large proportion of SIDS victims (2, 6, 7), raising the question of whether an underlying immunological deficiency contributes to these deaths. However, the potential role of inborn errors of immunity (IEI), including severe combined immunodeficiency (SCID), in SIDS has not been systematically investigated.

SCID is a rare, congenital immunodeficiency disorder where both cellular and humoral immunity are severely impaired due to the absence or dysfunction of T- and B-cells (8). Although infants with SCID appear healthy at birth, they are highly susceptible to infections, which can quickly progress to life-threatening conditions. SCID is often undiagnosed until severe infections or sepsis occur, frequently leading to death within the first year of life if not treated. The recognition of SCID and other IEI is challenging, and many cases likely remain undiagnosed (9), particularly in the absence of a comprehensive screening program. A recent report from the European Society for Immunodeficiencies, based on data from > 30.000 patients diagnosed with IEI, offers valuable insight into how genetic immune defects can manifest during early infancy (10). Although SIDS is not explicitly addressed in this report, the findings support the relevance of genetic screening into the investigation of unexplained infant deaths.

In this study, we explored whether SCID or other IEI could be implicated in cases of SIDS and undiagnosed infection-related deaths prior to the introduction of SCID screening in Norway on January 1, 2018. Given the complexity of diagnosing immune deficiencies and infections post-mortem, this study aims to shed light on the relationship between immune dysfunction and sudden infant and early childhood deaths.

## 2 Materials and methods

### 2.1 Study population and sources of data

Population data on number of deaths for the study period from 2012–2017 was obtained from Statistics Norway. Causes of death for the same period were obtained from the Norwegian Cause of Death Registry (CoDR), which encompasses all deaths within Norway and is based on death certificates. The registry has a stable coverage rate of 98%. Missing data primarily consists of deaths of Norwegian citizens occurring abroad (11). The validity of the data is ensured through several mechanisms, e.g. examination of diagnoses for plausibility based on the individual’s age and gender and inquiring the attending physician or institution if the underlying cause of death is unclear or missing. All autopsies performed are reported to the registry (11). For all deaths of children <1 year of age, a cross-reference of data with data from the Medical Birth Registry of Norway is performed.

The data obtained included demographic information of the deceased, time and cause of death classified according to the International Statistical Classification of Diseases and Health Related Problems, 10^th^ revision (ICD-10). Autopsy data were available for about 10% of all deaths. The underlying cause of death was determined according to the World Health Organization’s established guidelines. For the purposes of this study, all categories of cause of death, i.e. underlying, immediate, and contributing causes were regarded as equally significant. Infants were defined as individuals aged ≤2 years.

**Figure 1:**
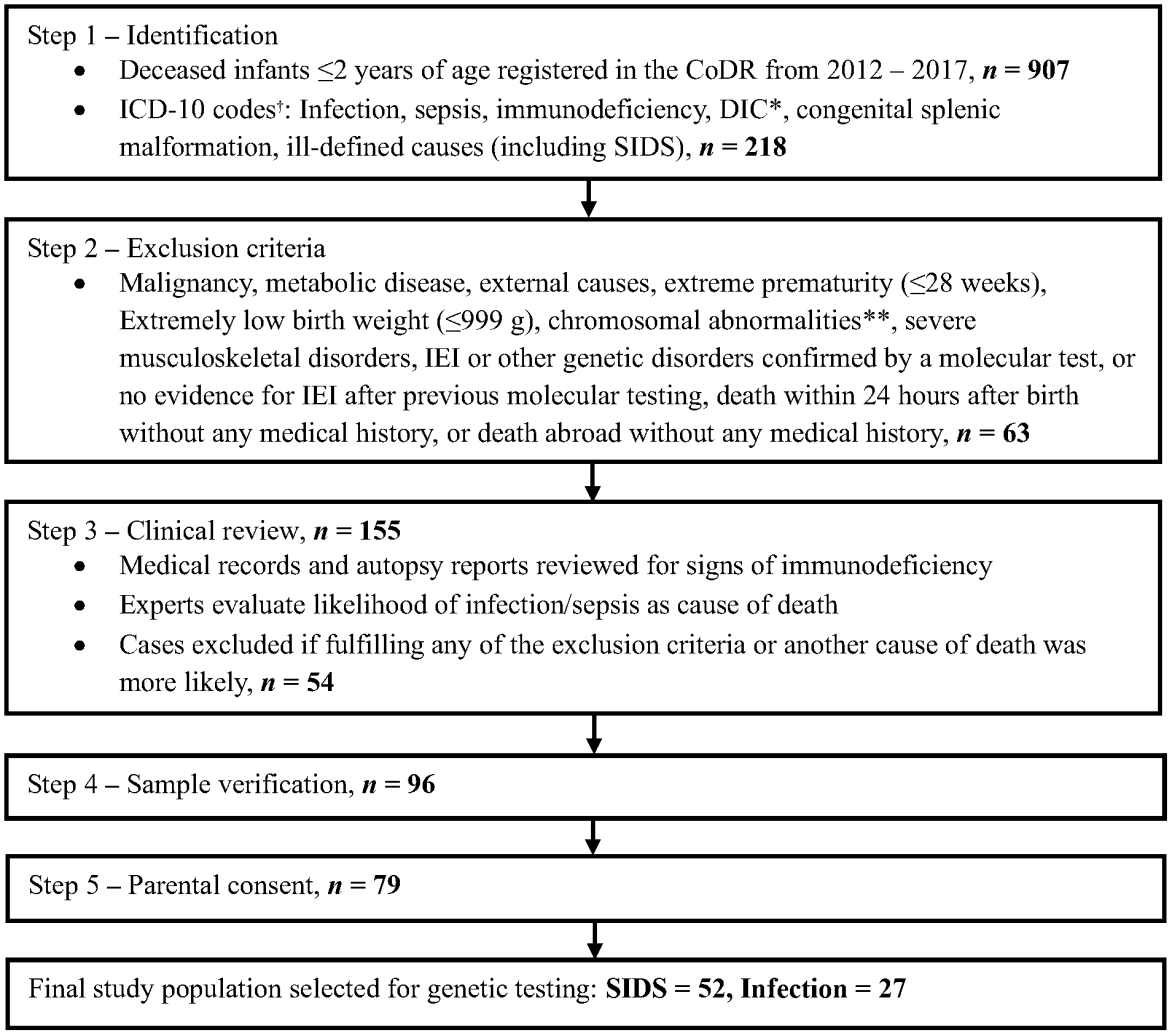
Study inclusion workflow. CoDR, Norwegian cause of death registry; DIC, disseminated intravascular coagulation; SIDS, sudden infant death syndrome; IEI, inborn error of immunity; ^†^A detailed overview of all the ICD-10 codes for inclusion is provided in **Supplementary Table 1**. *Two patients with DIC were excluded; one patient was affected by Rhesus isoimmunization (ICD-10=P55.0), the other was affected by placental transfusion syndrome (ICD-10=P02.3), both of which were considered as more likely causes of DIC rather than an undiagnosed infection. **One patient with Down's syndrome (ICD-10=Q90.9) and infection with Epstein-Barr virus (ICD-10=B27.0) was included as death caused by this virus is unusual.

The inclusion process (**Figure 1**) followed a five-step methodology: First, all infants who died before the age of 2 years during the study period and had an ICD-10 code for infection, sepsis, immunodeficiency, disseminated intravascular coagulation (DIC), congenital malformation of the spleen, or ill-defined and unspecified causes of mortality were identified (**Supplementary Table 1**).

Second, individuals were excluded if the cause of death was attributed to malignancy, metabolic disease, external causes, extreme prematurity (gestational age ≤28 weeks), extremely low birth weight (≤999 grams), chromosomal abnormalities, severe musculoskeletal disorders, IEI or other genetic disorders confirmed by a molecular test, or no evidence for IEI after previous molecular testing, death within 24 hours after birth without any medical history, or death abroad without any medical history. All inclusion/exclusion criteria were defined by consensus between pediatric immunology experts (TGA & HCEL).

Third, medical records and autopsy reports (when available) were reviewed for findings suggestive of immunodeficiency. The two experts jointly determined whether cases met predefined criteria indicating infection- or sepsis-related death. Infants were excluded if they met any exclusion criteria or if another cause of death was considered more likely. Consensus was required for genetic testing.

Fourth, for infants selected for genetic testing, the availability of biological samples was verified through the Norwegian National Newborn Screening (NBS) Unit at Oslo University Hospital (OUS). In Norway, dried blood spot samples are routinely collected from all newborns at 2–3 days of age, analyzed at the NBS Unit, and stored in a diagnostic biobank. Screening is performed with informed consent, and the collection, analysis, and storage of samples are regulated by national health legislation. For SIDS cases with post-mortem investigations conducted at the Department of Forensic Sciences at OUS, sample availability was verified through the department’s research biobank if no samples were available in the NBS.

Fifth, informed consent was obtained from the parents.

### 2.2 Analyses

The protocol for SCID/IEI screening is identical to the protocol currently employed by the NBS program for the detection of SCID in live-born infants (12). The original newborn screening filter card samples stored in the NBS research biobank were utilized for the analyses. First, a quantification of T-cell receptor excision circles (TREC) was performed using real-time quantitative polymerase chain reaction. TREC are circular DNA fragments produced during the formation of a normal T-cell receptor and serve as a surrogate marker for thymus function. Low levels of TREC are indicative of dysfunctional or absent T-cells. This method of TREC measurement is well-established and is highly sensitive and specific (13). In the ordinary newborn screening setting, TREC values below the cutoff threshold of ≤25/µl are classified as low, and values <5/µl urgently reported as suspected SCID. Sequencing was performed to identify the presence of variants associated with immunodeficiency in the study cohort.

Next, DNA was extracted and analyzed using Ion AmpliSeq library kit with an extended gene panel encompassing genes known to be associated with IEI and finally sequenced on a benchtop Ion GeneStudio S5 System (Thermo Fisher Scientific). List of genes included in the IEI gene panel is provided in the **Supplementary Table 2**.

All analyses were conducted at the NBS Unit’s laboratory at OUS. For five deceased children, blood samples collected during autopsy and stored in the research bank at the Department of Forensic Sciences at OUS were utilized for testing, as these children did not have available filter cards in the NBS.

Differences between groups were assessed using the chi-square test, with two-sided *p*-values <0.05 considered statistically significant. Gene variants were classified according to the American College of Medical Genomics and Genetics’ (ACMG) variant classification; RF=Risk factor, 1=benign; 2=likely benign; 3=variants of uncertain significance (VUS), 4=likely pathogenic, 5=pathogenic. Population allele frequencies were obtained from Genome Aggregation Database version 4.0 (GnomAD v4.0) (https://gnomad.broadinstitute.org). Variant interpretation was guided by ACMG/AMP 2015 criteria (14). Allele frequency was calculated for a subset of genes by dividing the number of observed variant alleles by the total number of alleles assessed at the locus. Each individual contributes two alleles unless otherwise specified.

## 3 Results

A total of 907 children aged ≤2 years died between 2012 and 2017, of which 54% were male. The median time to death was 7 days (interquartile range 68). Of these, 17% died from some form of infection, with the most common being perinatal infection and neonatal sepsis (9%), followed by respiratory infections (4%), other infections and sepsis (3%), and central nervous system infections (1%). Three children were registered with an immunodeficiency as the primary cause of death. Additionally, 9% of the deaths were attributed to ill-defined causes (ICD-10 codes: R95, R95.0, R96, R98, R99), including cases of SIDS.

Based on the ICD-10 codes on their death certificates, medical records for 155 deceased children were selected for review. After reviewing the medical records, 101 individuals were chosen for genetic testing. Of these, 36% (*n* = 36) had died from a recognized infection. Samples for TREC testing and genetic analyses were available for 95% (*n* = 96) of the eligible children; however, parental consent for testing was not achieved for 17 cases, including nine children who died from a recognized infection. Thus, the final study population consisted of 79 cases, of which 66% (*n* = 52) died of SIDS and 34% (*n* = 27) due to infection.

The majority in both groups was males (67%) and died before 4 months of age; 52% (*n* = 27) for the SIDS group, and 67% (*n* = 18) for the infection group. Preterm birth was observed in 19% (*n* = 10) of infants within the SIDS group, compared to 33% (*n* = 9) among those in the infection group. Mean TREC levels in both groups were >200/µl. Lowest TREC level detected was 22/µl (range 22–1060/µl). This infant died from an infection, and genetic screening revealed a VUS in an autosomal dominant gene. A summary of all gene variants detected with corresponding demographic, clinical and immunological characteristics is provided in **Table 1**.

**Table 1:** Demographic, immunological and genetic characteristics of 79 deceased infants ≤2 years old in Norway from 2012 to 2017.

Variables	Total n = 79 (100%)	SIDS n = 52 (66%)	Infection n = 27 (34%)
Median age, days (IqR)	80 (229)	117 (237)	51 (412)
Age at death <120 days	45 (57)	27 (52)	18 (67)
Prematurity, gestational age <37 weeks	19 (24)	10 (19)	9 (33)
Males	53 (67)	35 (67)	18 (67)
Median TREC, µl (IqR)	250 (150)	267 (186)	208 (139)
All infants with at least one gene variant	55 (70)	35 (67)	20 (74)
*MBL2* variants	33 (42)	19 (37)	14 (52)
VUS in autosomal dominant disease genes*	14 (18)	13 (25)	1 (4)
One pathogenic variant in autosomal recessive disease genes**	18 (23)	8 (15)	10 (37)

SIDS, sudden infant death syndrome; IqR, interquartile range; TREC, T-cell exicion circles; MBL, mannose-binding lectin; VUS, variants of uncertain significance; *None of the variants were clearly pathologic; **Including individuals with variants in >1 genes; parentheses are percentages of the total in each group unless specified otherwise.

In both groups, the majority of individuals were found to carry at least one genetic IEI-related variant, 67% (*n* = 35) for the SIDS group, and 74% (*n* = 20) for the infection group. None of the identified genotypes were clearly pathogenic. VUS in autosomal dominant disease genes were identified in 18% (*n* = 14) of the cohort (**Supplementary Table 3**). Heterozygous pathogenic variants (ACMG class 5) in autosomal recessive genes were detected in 23% (*n* = 18) of the cohort, including cases with variants in more than one gene (**Table 2**). Genetic variants classified as ACMG RF were identified in 42% (*n* = 33) of the cohort (**Table 3**).

**Table 2:** Pathogenic variants classified as ACMG 5 in autosomal recessive genes and other characteristics for 18 individuals.

Pt #	Cause of death	GA (w)	Birth weight (kg)	Sex	Age (w)	TREC (μl)	Sudden death (<24h)	Significant findings	Gene	Reference sequence	Inh IEI	Variant	GnomAD v4.0
4	SIDS	35	2	Boy	6	200	Yes	None	*TNFRSF13B (TACI)*	NM_012452.3	AD/AR	c.542C>A, p.Ala181Glu	0.0054
5	SIDS	38	3	Girl	3	215	Yes	*na*	*C9*	NM_001737.5	AR	c.162C>A, p.Cys54*	0.0012
6	SIDS	40	3	Boy	3	215	Yes	None	*SPINK5*	NM_006846.4	AR	c.891C>T, splice defect	5.10e-05
7	SIDS	39	3	Girl	8	352	Yes	None	*C2*	NM_000063.6	AR	c.841_849+19del, p.Val281_Arg283del	0.0057
14	SIDS	40	4	Girl	9	182	Yes	None	*AIRE*	NM_000383.4	AR (LOF)	c.1249_1250insC, p.Leu417Profs*7	1.52e-05
17	SIDS	40	3	Girl	77	295	Yes	None	*CD247*	NM_198053.3	AR	c.301C>T, p.Gln101*	0.00027
19	SIDS	39	3	Boy	24	224	Yes	None	*TNFRSF13B (TACI)*	NM_012452.3	AD/AR	c.542C>A, p.Ala181Glu	0.0054
55	SIDS	37	3	Boy	16	125	Yes	Thymus hypoplasia	*C6*	NM_000065.5	AR	c.1352dupA, p.Tyr451*	0.0001
3	Infection	38	4	Girl	61	268	Yes	*Haemophilus influenzae* pneumonia	*UNC13D*	NM_199242.3	AR	c.2695C>T, p.Arg899*	1.35e-05
10	Infection	42	4	Boy	4	33	*na*	*na*	*CARMIL2*	NM_001013838.3	AR	c.245de1,p.Pro82Leufs*127	0
11	Infection	30	1	Boy	3	366	*na*	Septicemia	*SKIC3 (TTC37)*	NM_014639.4	AR	c.3625C>T, p.Arg1209*	4.34e-05
15	Infection	41	4	Girl	9	216	No	Extensive lung fibrosis, purulent tracheobronchitis	*C8A*	NM_000562.3	AR	c.1492C>T, p.Arg498*	1.36e-05
16	Infection	38	2	Boy	18	42	Yes	Unbalanced chromosomal aberration	*G6PD*	NM_001360016.2	XL	c.563C>T, p.Ser188Phe	0.0014
									*FCN3*	NM_003665.4	AR	c.70C>T, p.Gln24*	8.05e-06
22	Infection	40	4	Boy	53	170	*na*	*Streptococcus pyogenes* septicemia	*C9*	NM_001737.5	AR	c.162C>A, p.Cys54*	0.0012
23	Infection	31	2	Boy	2	172	*na*	*na*	*ZAP70*	NM_001079.4	AR	c.747C>A, p.Cys249*	1.59e-06
24	Infection	39	3	Girl	71	160	No	Necrotizing encephalopathy	*GBA*	NM_000157.4	AR	c.1226A>G, p.Asn409Ser	0.001997
25	Infection	35	2	Girl	79	28	*na*	Bacterial pneumonia	*STAT5B*	NM_012448.4	AR (LOF)	c.616C>T, p.Gln206*	0
31	Infection	*na*	*na*	Girl	68	*np*	No	*Haemophilus influenzae* Acute pneumonia	*TMC8*	NM_152468.5	AR	c.1481del, p.Leu494Argfs*5	0.00025

Pt, patient; w, week; kg, kilogram; h, hour; TREC, T-cell receptor exicion circles; GA, gestational age; GnomAD v4.0, genome aggregation database version 4.0; Inh, inheritance; IEI, inborn errors of immunity; SIDS, sudden infant death syndrome; AD, autosomal dominant; AR, autosomal recessive; LOF, loss-of-function variant; na, not available; RF, risk factor; VUS, variants of uncertain significance; XL, X-linked; 2, likely benign 3, VUS; 4, likely pathogenic; 5, pathogenic; np, not performed.(*) indicates a translation termination codon (stop codon) according to HGVS nomenclature.

**Table 3:** Gene variants classified as ACMG risk factor and other characteristics for 33 individuals.

Pt #	Cause of death	GA (w)	Birth weight (kg)	Sex	Age (w)	Sudden death (<24h)	TREC (μl)	Significant findings	Gene	Reference sequence	Inh IEI	Variant	GnomAD v4.0
4	SIDS	35	2	Boy	6	Yes	200	None	*MBL2*	NM_001378373.1	AD	c.161G>A, p.Gly54Asp	0.139
9	SIDS	40	4	Girl	19	Yes	246	None	*MBL2*	NM_001378373.1	AD	c.154C>T(;)161G>A, p.Arg52Cys(;)Gly54Asp	0.064(;)0.139
13	SIDS	36	2	Boy	11	Yes	189	Lymphoid interstitial pneumonia	*MBL2*	NM_001378373.1	AD	c.161G>A, p.Gly54Asp	0.139
14	SIDS	40	4	Girl	9	Yes	182	None	*MBL2*	NM_001378373.1	AD	c.161G>A, p.Gly54Asp	0.139
28	SIDS	36	3	Boy	5	Yes	267	None	*MBL2*	NM_001378373.1	AD	c.161G>A, p.Gly54Asp	0.139
35	SIDS	32	2	Boy	3	Yes	82	Elevated cotinine levels	*MBL2*	NM_001378373.1	AD	c.154C>T, p.Arg52Cys	0.064
36	SIDS	40	3	Boy	8	Yes	285	None	*MBL2*	NM_001378373.1	AD	c.154C>T, p.Arg52Cys	0.064
38	SIDS	40	4	Boy	6	Yes	329	None	*MBL2*	NM_001378373.1	AD	c.154C>T, p.Arg52Cys	0.064
40	SIDS	41	3	Girl	0	Yes	198	None	*MBL2*	NM_001378373.1	AD	c.161G>A, p.Gly54Asp	0.139
41	SIDS	34	2	Boy	15	Yes	145	None	*MBL2*	NM_001378373.1	AD	c.161G>A, p.Gly54Asp	0.139
42	SIDS	36	3	Boy	43	Yes	203	None	*MBL2*	NM_001378373.1	AD	c.161G>A, p.Gly54Asp	0.139
43	SIDS	39	3	Boy	8	Yes	279	Elevated alloisoleucine	*MBL2*	NM_001378373.1	AD	c.161G>A, p.Gly54Asp	0.139
44	SIDS	36	3	Boy	16	Yes	231	None	*MBL2*	NM_001378373.1	AD	c.161G>A, p.Gly54Asp	0.139
45	SIDS	*na*	*na*	Boy	75	Yes	np	None	*MBL2*	NM_001378373.1	AD	c.161G>A, p.Gly54Asp	0.139
53	SIDS	38	3	Boy	13	Yes	250	None	*MBL2*	NM_001378373.1	AD	c.154C>T, p.Arg52Cys	0.064
55	SIDS	37	3	Boy	16	Yes	125	Thymus hypoplasia	*MASP2*	NM_006610.4	AR	c.1921G>A, p.Asp641Asn	0.0002
3	Infection	38	4	Girl	61	Yes	268	*Haemophilus influenzae* pneumonia	*MBL2*	NM_001378373.1	AD	c.161G>A, p.Gly54Asp	0.139
10	Infection	42	5	Boy	4	*na*	33	*na*	*MBL2*	NM_001378373.1	AD	c.161G>A, p.Gly54Asp	0.139
15	Infection	41	4	Girl	9	No	216	Extensive lung fibrosis, purulent tracheobronchitis	*MBL2*	NM_001378373.1	AD	c.154C>T, p.Arg52Cys	0.064
22	Infection	40	4	Boy	53	*na*	170	*Streptococcus pyogenes* septicemia	*MBL2*	NM_001378373.1	AD	c.154C>T, p.Arg52Cys	0.064
23	Infection	31	2	Boy	2	*na*	172	Septicemia	*MBL2*	NM_001378373.1	AD	c.154C>T, p.Arg52Cys	0.064
25	Infection	35	2	Girl	79	*na*	28	Bacterial pneumonia	*MBL2*	NM_001378373.1	AD	c.154C>T(;)161G>A, p.Arg52Cys(;)Gly54Asp	0.064(;)0.139
32	Infection	39	4	Boy	1	*na*	180	*na*	*MBL2*	NM_001378373.1	AD	c.154C>T, p.Arg52Cys	0.064
33	Infection	31	2	Boy	2	Yes	125	Multidrug-resistant *Escherichia coli* septicemia	*MBL2*	NM_001378373.1	AD	c.154C>T, p.Arg52Cys	0.064
34	Infection	30	2	Boy	5	*na*	106	Bacterial pneumonia	*MBL2*	NM_001378373.1	AD	c.154C>T, p.Arg52Cys	0.064
37	Infection	39	3	Boy	1	Yes	499	*Group B Streptococcus* septicemia	*MBL2*	NM_001378373.1	AD	c.154C>T, p.Arg52Cys	0.064
39	Infection	36	3	Girl	2	Yes	229	*Group B Streptococcus* septicemia	*MBL2*	NM_001378373.1	AD	c.154C>T, p.Arg52Cys	0.064
46	Infection	37	4	Boy	78	Yes	121	*na*	*MBL2*	NM_001378373.1	AD	c.161G>A;(161G>A), p.Gly54Asp;(Gly54Asp)	0.139
47	Infection	41	3	Boy	4	Yes	517	*Group B Streptococcus* septicemia	*MBL2*	NM_001378373.1	AD	c.161G>A;(161G>A), p.Gly54Asp;(Gly54Asp)	0.139
48	Infection	29	1	Boy	7	*na*	192	*Cytomegalovirus* viremia	*MBL2*	NM_001378373.1	AD	c.161G>A;(161G>A), p.Gly54Asp;(Gly54Asp)	0.139

Pt, patient; GA, gestational age; w, week; kg, kilogram; h, hour; TREC, T-cell receptor exicion circles; µl, microliter; Inh, inheritance; IEI, inborn errors of immunity; GnomAD v4.0, genome aggregation database version 4.0; SIDS, sudden infant death syndrome; AD, autosomal dominant; AR, autosomal recessive; na, not available; np, not performed.

**Table 4** shows the allele frequency for mannose-binding lectin 2 (*MBL2*) variants for the two groups. Two of the variants were classified as ACMG RF, and one as likely benign (ACMG class 2). Within the infection group, the variants c.154C>T, p.Arg52Cys and c.161G>A, p.Gly54Asp (both ACMG RF), were detected in 9 out of 54 alleles, yielding an allele frequency of 0.17 (17%). The allele frequency of p.Arg52Cys was significantly higher than that observed in the general population (6%) (*p =* 0.02, 95% CI). The frequency of p.Gly54Asp was higher than in the general population (14%) but this difference was not significant. The allele frequencies of these two variants in the SIDS group were comparable to those found in the general population (5 and 11%, respectively).

**Table 4:** Allele frequencies of mannose-binding lectin 2 variants.

Gene	Variant on cDNA level	Protein effect	ACMG	GnomAD v4.0	SIDS variant/ total alleles	*p*	Infection variant/ total alleles	*p*
*MBL2*	c.154C>T	p.Arg52Cys	RF	0.06	5/104 = 0.05	ns	9/54 = 0.17	0.02
*MBL2*	c.161G>A	p.Gly54Asp	RF	0.14	11/104 = 0.11	ns	9/54 = 0.17	ns
*MBL2*	c.170G>A	p.Gly57Glu	2	0.03	5/104 = 0.08	ns	0	NA

ACMG, The American College of Medical Genetics and Genomics’ variant classification; GnomAD v4.0, genome aggregation database version 4.0; SIDS, sudden infant death syndrome; *MBL2*, mannose-binding lectin 2; RF, risk factor; 2, likely benign; *p*, *p*-*value*; ns, not significant; NA, not applicable. The *p-value* in this table refers to the difference in allele frequencies between the general population and the respective cohorts.

## 4 Discussion

In this nationwide, population-based study, encompassing all deceased children ≤2 years of age over a period of 6 years prior to the implementation of SCID screening in Norway, we did not find any missed cases of SCID or IEI among infants who died from infections or SIDS. Nevertheless, three deceased infants with SCID/IEI were identified through the CoDR during this period. These were not included in our cohort as their diagnoses were already established, albeit too late to prevent mortality. These cases underline the diagnostic challenges, as recognized by Stray-Pedersen et al. over two decades ago (9). In their study, four out of five SCID infants in Norway died before a diagnosis could be made, primarily due to infections that could have been prevented with earlier detection. The absence of systematic newborn screening for SCID at the time was a major factor in these missed diagnoses, leading to fatalities.

Research exploring the genetic basis of SIDS is limited (3), evidence suggests that immune dysregulation may contribute to vulnerability in a subset of cases (15). To the best of our knowledge, our study is among the first to investigate the potential role of IEI in SIDS from a genetic perspective in a national cohort. SIDS is widely recognized as a multifactorial condition, with established risk factors including prone sleeping position, parental smoking, prematurity, and recent mild infections (2). In a subset of our SIDS cohort, clinical signs consistent with infection were observed prior to death, and post-mortem examinations frequently revealed pathological findings indicative of infectious processes, as previously reported (3, 5, 16, 17). However, post-mortem microbiological results often present diagnostic challenges, and pathognomonic findings remain elusive (16, 18).

Although we did not identify any missed cases of SCID or other IEI among infants who died from infections or SIDS, a significant proportion of the cohort may carry genetic predispositions, with pathogenic autosomal recessive variants present in about one-quarter and risk-related variants in nearly half of the individuals. The implications of these findings are not yet known, but their presence could indicate a genetically mediated predisposition to immune imbalance or impaired pathogen handling. Genetic variants affecting immune pathways, particularly those involved in innate immunity, inflammation, and host-pathogen interactions, could potentially contribute to this vulnerability (19). An immune dysregulation, such as abnormal cytokine responses or subclinical infection, could act as precipitating factors in infants with underlying vulnerabilities (2, 20). Finally, these findings must be interpreted in the context of known SIDS risk factors, i.e. there might be an interaction between the presence of immune-related pathogenic variants and environmental and developmental factors, thus lowering the threshold for fatal outcomes. For example, an infant with a mild respiratory infection and a genetic vulnerability towards reduced immune responsiveness, may be less able to mount an effective defense, especially in the presence of other stressors such as prone sleeping or tobacco exposure (2).

MBL plays a key role in the lectin pathway of complement activation, facilitating opsonization and clearance of pathogens (21). Deficiency or functional impairment due to variants in the *MBL2* gene, has been associated with increased susceptibility to infections, notably in early childhood when adaptive immunity is still developing (22). MBL deficiency alone is not typically sufficient to cause severe disease in otherwise healthy individuals but could possibly act as a risk-modifying factor in the presence of other stressors, such as co-infection, prematurity, or environmental exposures. The higher prevalence of *MBL2* variants in infants with infection-related deaths compared with the general population represents an observed difference in genetic distribution. At present, the relevance of these innate immune variants to infection-related mortality in early childhood remains undetermined. The findings may serve to contextualize broader hypotheses concerning immune dysregulation or hyperinflammatory responses in SIDS or deaths following unrecognized infections.

TREC levels, gestational age and birth weight were comparable across the two cohorts. TREC levels are commonly used as a proxy for naïve T-cell production. The absence of differences in TREC levels indicate that severe T-cell lymphopenia is unlikely to be a contributing factor in either group. Moreover, the lack of differences in gestational age and birth weight reduces the likelihood that prematurity or intrauterine growth restriction played a significant role in vulnerability to mortality in the two cohorts. These findings support the concept that other immunological or genetic factors may be more relevant in explaining susceptibility in these cases.

Male infants are typically overrepresented in both cases of SIDS (55–60% of cases) and infection-related mortality among infants (23, 24). Differences in sex-specific immune modulation, hormonal influences, and dysregulated inflammatory responses are postulated as explanatory factors (25). The proportion of male deaths in our study was higher than previously reported, with 67% of both SIDS and infection-related deaths occurring in boys. The elevated proportion could possibly indicate an underlying difference in genetic susceptibility and a particular vulnerability within our cohort.

Despite the strengths of our study, including the use of a national population-based registry with high completeness and validity, a thorough review of the medical history and the application of a well-established method for detecting IEI, certain limitations exist. In the context of SIDS, data on known risk factors are lacking, limiting the ability to assess the interaction between potential genetic predisposition and environmental triggers. Medical history was reviewed retrospectively, and relevant variables such as prior infections and family history may be under-reported or missing. Autopsy data were available for only a limited number of cases, constraining a further systematic assessment of possible infectious findings. The strict exclusion criteria may have resulted in an underestimation of SCID in preterm or syndromic infants (26, 27). Also, based on the current methodology used for genetic screening in our study, we cannot rule out the possibility that other pathogenic variants might be identified if more advanced techniques were applied, such as whole genome analysis, which could potentially reveal structural variants like deletions. Furthermore, the number of genes associated with primary immunodeficiencies is constantly increasing, and we may have missed a genetic diagnosis due to limitations in the gene panel tested. Finally, genetic testing without parental consent was not accepted by national ethical regulations. Although parental consent was obtained for the majority of cases, a small number of parents did not consent to genetic testing despite our best efforts in ensuring anonymization of the patients. The impact of this on the overall findings remains unclear, particularly for the nine missing cases who had known infections. Whether any of these cases involved undiagnosed SCID or IEI is thus unknown.

Our study provides valuable insights into the genetic basis of SIDS and infection deaths in early life, two major contributors to post-neonatal infant mortality. Our findings are in agreement with the multifactorial model of SIDS, in which a vulnerable infant encounters a critical stressor during a sensitive developmental window. For both groups, the presence of immune-related genetic variants, even in the absence of overt immunodeficiency, may reflect a latent vulnerability that interacts with physiological and environmental factors. Future research should incorporate registry-based epidemiological data to enable matched control comparisons and familial risk assessment and further explore the gene-environment interactions. Such efforts are essential to elucidate the complex interplay between genetic susceptibility and environmental exposures in early childhood mortality and may ultimately form preventive strategies and early identification of at-risk infants.

## Data Availability

The datasets presented in this article are not readily available because of the Norwegian GDP-rules. Requests to access the datasets should be directed to the corresponding author.

## Supplementary material

Supplementary Table 1 ICD-10 codes for inclusion.

Supplementary Table 2 Genes included in the next generation sequencing immunodeficiency panel copy number variation (366 genes).

Supplementary Table 3 Characteristics of 79 deceased infants.
