# Supplementary material for: Unmasking immunodeficiency in sudden infant deaths: no evidence of hidden immune disorders
Source: Front Immunol. 2026 Aug 5;17:1860011. doi: 10.3389/fimmu.2026.1860011 (PMC13485708; doi:10.3389/fimmu.2026.1860011)
Supplement: Supplementary file 1 [file Table1.docx]

| **Supplementary table 1**. ICD-10 codes for inclusion. | | | | | | |  |
| --- | --- | --- | --- | --- | --- | --- | --- |
|  | |  | | |  |  |  |
| **ICD-10 codes** | |  | | | **Title** |  |  |
| A08.0 | |  | | | Rotaviral enteritis | |  |
| A08.1 | |  | | | Acute gastroenteropathy due to Norwalk agent | |  |
| A39.2 | |  | | | Acute meningococcaemia | |  |
| A40.0 | |  | | | Sepsis due to streptococcus, group A | |  |
| A40.1 | |  | | | Sepsis due to streptococcus, group B | |  |
| A40.3 | |  | | | Sepsis due to Streptococcus pneumoniae | |  |
| A41.0 | |  | | | Sepsis due to Staphylococcus aureus | |  |
| A41.5 | |  | | | Sepsis due to other Gram-negative organisms | |  |
| A41.9 | |  | | | Sepsis, unspecified | |  |
| A49.8 | |  | | | Other bacterial infections of unspecified site | |  |
| B00.4 | |  | | | Herpesviral encephalitis | |  |
| B08.2 | |  | | | Exanthema subitum [sixth disease] | |  |
| B27.0 | |  | | | Gammaherpesviral mononucleosis - Mononucleosis due to Epstein-Barr virus | |  |
| B34.9 | |  | | | Viral infection, unspecified | |  |
| B37.7 | |  | | | Candidal sepsis | |  |
| B44.9 | |  | | | Aspergillosis, unspecified | |  |
| B99 | |  | | | Other and unspecified infectious diseases | |  |
| D81.8 | |  | | | Other combined immunodeficiencies; Biotin-dependent carboxylase deficiency | |  |
| D82.1 | |  | | | Di George syndrome | |  |
| D84.9 | |  | | | Immunodeficiency, unspecified | |  |
| G00.2 | |  | | | Streptococcal meningitis | |  |
| G00.3 | |  | | | Staphylococcal meningitis | |  |
| G00.8 | |  | | | Other bacterial meningitis | |  |
| G03.9 | |  | | | Meningitis, unspecified | |  |
| J06.9 | |  | | | Acute upper respiratory infection, unspecified | |  |
| J12.1 | |  | | | Respiratory syncytial virus pneumonia | |  |
| J12.2 | |  | | | Parainfluenza virus pneumonia | |  |
| J12.3 | |  | | | Human metapneumovirus pneumonia | |  |
| J12.8 | |  | | | Other viral pneumonia | |  |
| J12.9 | |  | | | Viral pneumonia, unspecified | |  |
| J14 | |  | | | Pneumonia due to Haemophilus influenzae | |  |
| J18.0 | |  | | | Bronchopneumonia, unspecified | |  |
| J18.9 | |  | | | Pneumonia, unspecified | |  |
| J20.9 | |  | | | Acute bronchitis, unspecified | |  |
| J21.0 | |  | | | Acute bronchiolitis due to respiratory syncytial virus | |  |
| J22 | |  | | | Unspecified acute lower respiratory infection | |  |
| P23.0 | |  | | | Congenital pneumonia due to viral agent | |  |
| P23.6 | |  | | | Congenital pneumonia due to other bacterial agents | |  |
| P23.9 | |  | | | Congenital pneumonia, unspecified | |  |
| P35.1 | |  | | | Congenital cytomegalovirus infection | |  |
| P35.2 | |  | | | Congenital herpesviral [herpes simplex] infection | |  |
| P35.8 | |  | | | Other congenital viral diseases | |  |
| P36.0 | |  | | | Sepsis of newborn due to streptococcus, group B | |  |
| P36.2 | |  | | | Sepsis of newborn due to Staphylococcus aureus | |  |
| P36.4 | |  | | | Sepsis of newborn due to Escherichia coli | |  |
| P36.5 | |  | | | Sepsis of newborn due to anaerobes | |  |
| P36.8 | |  | | | Other bacterial sepsis of newborn | |  |
| P36.9 | |  | | | Bacterial sepsis of newborn, unspecified | |  |
| P39.9 | |  | | | Infection specific to the perinatal period, unspecified | |  |
| D65 |  | | | Disseminated intravascular coagulation [defibrination syndrome] | | | |
| P60 |  | | | Disseminated intravascular coagulation of fetus and newborn | | | |
| Q89.0 |  | | | Congenital malformations of spleen - Asplenia (congenital), Congenital splenomegaly | | | |
| R95 |  | | | Sudden infant death syndrome | | | |
| R95.0 |  | | | Sudden infant death syndrome with mention of autopsy | | | |
| R96 |  | | | Other sudden death, cause unknown | | | |
| R98 |  | | | Unattended death | | | |
| R99 |  | | | Other ill-defined and unspecified causes of mortality | | | |
|  | | |  | | | | |
