# Supplementary material for: Unmasking immunodeficiency in sudden infant deaths: no evidence of hidden immune disorders
Source: Front Immunol. 2026 Aug 5;17:1860011. doi: 10.3389/fimmu.2026.1860011 (PMC13485708; doi:10.3389/fimmu.2026.1860011)
Supplement: Supplementary file 2 [file Table2.docx]

**Supplementary table 2**. Genes included in the Next generation Sequencing immunodeficiency panel copy number variation (366 genes).

| ACP5 | CD3G | EPCAM | IL12RB1 | LIG4 | NHP2 | RIPK1 | STAT1 | TTC37 |
| --- | --- | --- | --- | --- | --- | --- | --- | --- |
| ADA | CD40 | EPG5 | IL12RB2 | LIPA | NLRC4 | RLTPR | STAT2 | TTC7A |
| ADAM17 | CD40LG | ERAP1 | IL17F | LMBRD1 | NLRP1 | RNASEH2A | STAT3 | TYK2 |
| ADAR | CD55 | EXTL3 | IL17RA | LPIN2 | NLRP12 | RNASEH2B | STAT4 | UNC119 |
| AICDA | CD70 | FAAP24 | IL17RC | LRBA | NLRP3 | RNASEH2C | STAT5B | UNC13D |
| AIRE | CD79A | FADD | IL1RN | LRRC8A | NOD2 | RNF168 | STIM1 | UNC93B1 |
| AK2 | CD79B | FAS | IL21 | LYST | NOP10 | RNF31 | STK4 | UNG |
| ANO6 | CD81 | FASLG | IL21R | MAGT1 | NRAS | RORC | STX11 | USB1 |
| APOL1 | CDC42 | FAT4 | IL2RA | MALT1 | OAS1 | RTEL1 | STXBP2 | USP18 |
| ARPC1B | CDCA7 | FBXW7 | IL2RB | MAN2B1 | OSTM1 | RUNX1 | TAP1 | USP43 |
| ATM | CEBPE | FCGR3A | IL2RG | MAP3K14 | PARN | SALL4 | TAP2 | VPS13B |
| ATP6V0A2 | CECR1 | FCN3 | IL36RN | MASP1 | PGM3 | SAMD9 | TAPBP | VPS45 |
| B2M | CFB | FECH | IL6 | MASP2 | PIK3CD | SAMD9L | TAZ | WAS |
| B3GALT4 | CFD | FN1 | IL6ST | MBL2 | PIK3R1 | SAMHD1 | TCF3 | WDR1 |
| B4GALT1 | CFH | FOXN1 | IL7R | MBTPS2 | PLCG2 | SBDS | TCIRG1 | WIPF1 |
| BACH2 | CFI | FOXP3 | INO80 | MCM10 | PLEKHM1 | SEC61A1 | TERF1 | WRAP53 |
| BCL10 | CFP | FTMT | IRAK4 | MCM4 | PNP | SEMA3E | TERT | XIAP |
| BLNK | CHD7 | G6PC | IRF1 | MED12 | POLA1 | SERPING1 | TFRC | XK |
| BTK | CIITA | G6PC3 | IRF2BP2 | MEFV | POLE | SFTPA1 | TGFB1 | XRCC4 |
| C1QA | CLCN7 | G6PD | IRF3 | MIF | POMP | SFTPA2 | TICAM1 | ZAP70 |
| C1QB | CLEC7A | GATA2 | IRF7 | MKL1 | PRF1 | SH2D1A | TINF2 | ZBTB24 |
| C1QC | COLEC11 | GBA | IRF8 | MMRN1 | PRKCD | SHARPIN | TIRAP | ZNF341 |
| C1R | COPA | GFI1 | ISG15 | MOGS | PRKDC | SKIV2L | TLR3 |  |
| C1S | COQ2 | GTF2H5 | ITCH | MRE11A | PRNP | SLC11A1 | TMC6 |  |
| C2 | CORO1A | HAX1 | ITGA2 | MS4A1 | PSENEN | SLC22A3 | TMC8 |  |
| C3 | CR2 | HELLS | ITGB2 | MSN | PSMB8 | SLC25A19 | TMEM173 |  |
| C5 | CSF3R | HMOX1 | ITGB3 | MST1 | PSTPIP1 | SLC25A33 | TMPRSS6 |  |
| C6 | CTLA4 | HOXA11 | ITK | MTHFD1 | PTCRA | SLC25A37 | TNFAIP3 |  |
| C7 | CTPS1 | ICOS | JAGN1 | MVK | PTEN | SLC29A3 | TNFRSF11A |  |
| C8A | CTSC | ICOSLG | JAK3 | MYD88 | PTPRC | SLC35A1 | TNFRSF11B |  |
| C8B | CXCR4 | IFIH1 | KCNA4 | MYO5A | RAB27A | SLC35C1 | TNFRSF13B |  |
| C9 | CYBA | IFNAR1 | KDM6A | MYSM1 | RAC2 | SLC37A4 | TNFRSF13C |  |
| CARD11 | CYBB | IFNAR2 | KIT | NBAS | RAG1 | SLC4A1 | TNFRSF1A |  |
| CARD14 | DCLRE1B | IFNG | KITLG | NBN | RAG2 | SMARCAL1 | TNFRSF4 |  |
| CARD9 | DCLRE1C | IFNGR1 | KLF1 | NCF1 | RASGRP1 | SMARCD2 | TNFRSF9 |  |
| CASP10 | DIAPH1 | IFNGR2 | KLKB1 | NCF2 | RBCK1 | SNX10 | TNFSF11 |  |
| CASP8 | DKC1 | IGLL1 | KMT2D | NCF4 | REL | SOCS1 | TNFSF12 |  |
| CCBE1 | DNMT3B | IKBKB | KRAS | NCSTN | RELA | SP110 | TPP1 |  |
| CD19 | DOCK2 | IKZF1 | LAMTOR2 | NFAT5 | RELB | SPI1 | TPP2 |  |
| CD247 | DOCK8 | IL10 | LAT | NFKB1 | RFX5 | SPINK5 | TRAF3 |  |
| CD27 | ELANE | IL10RA | LCAT | NFKB2 | RFXANK | SPPL2A | TRAF3IP2 |  |
| CD3D | EOMES | IL10RB | LCK | NFKBIA | RFXAP | SRP54 | TREX1 |  |
| CD3E | EP300 | IL12B | LIG1 | NHEJ1 | RHOH | SRP72 | TRNT1 |  |
