# Supplementary material for: Unmasking immunodeficiency in sudden infant deaths: no evidence of hidden immune disorders
Source: Front Immunol. 2026 Aug 5;17:1860011. doi: 10.3389/fimmu.2026.1860011 (PMC13485708; doi:10.3389/fimmu.2026.1860011)
Supplement: Supplementary file 3 [file Table3.docx]

| **Pt #** | **Cause of death** | **T REC**  **(µl)** | **GA (w)** | **Birth weight (kg)** | **Sex** | **Age (w)** | **Autopsy** | **Sudden death (<24h)** | **Gene** | | **Variant** | **ACMG** | **GnomAD v4.0** | **Reference sequence** | **Inh**  **IEI** | |
| --- | --- | --- | --- | --- | --- | --- | --- | --- | --- | --- | --- | --- | --- | --- | --- | --- |
| 1 | SIDS | 414 | 32 | 1 | Girl | 43 | Forensic | Yes | ***PLCG2*** | | c.2238A>C, p.Glu746Asp | 3 | 0 | NM_002661.5 | AD | |
| 2 | SIDS | 192 | 40 | 3 | Boy | 53 | Forensic | Yes | ***PIK3R1*** | | c.1925G>A, p.Arg642Gln | 3 | 1.30e-05 | NM_181523.3 | AD/AR | |
| 2 | SIDS |  |  |  |  |  |  |  | ***DOCK2*** | | c.1426T>C, p.Tyr476His | 3 | 5.89e-05 | NM_004946.3 | AR | |
| 4 | SIDS | 200 | 35 | 2 | Boy | 6 | Forensic | Yes | ***TNFRSF13B (TACI)*** | | c.542C>A, p.Ala181Glu | 5 | 0.0054 | NM_012452.3 | AD/AR | |
| 4 | SIDS |  |  |  |  |  |  |  | ***MBL2*** | | c.161G>A, p.Gly54Asp | RF | 0.139 | NM_001378373.1 | AD | |
| 5 | SIDS | 215 | 38 | 3 | Girl | 3 | Forensic | Yes | ***PIK3CD*** | | c.1642C>T, p.Arg548Trp | 3 | 3.73e-05 | NM_005026.5 | AD/AR | |
| 5 | SIDS |  |  |  |  |  |  |  | ***SAMD9*** | | c.1831C>T, p.Leu611Phe | 3 | 6.41e-06 | NM_017654.4 | AD/som | |
| 5 | SIDS |  |  |  |  |  |  |  | ***C9*** | | c.162C>A, p.Cys54* | 5 | 0.0012 | NM_001737.5 | AR | |
| 6 | SIDS | 215 | 40 | 3 | Boy | 3 | Forensic | Yes | ***SPINK5*** | | c.891C>T, splice defect | 5 | 5.10e-05 | NM_006846.4 | AR | |
| 7 | SIDS | 352 | 39 | 3 | Girl | 8 | Forensic | Yes | ***IFIH1*** | | c.2788A>C, p.Asn930His | 3 | 5.85e-05 | NM_022168.4 | AD | |
| 7 | SIDS |  |  |  |  |  |  |  | ***SPINK5*** | | c.775_776delGCinsTT, p.Ala259Phe | 3 | 0 | NM_006846.4 | AR | |
| 7 | SIDS |  |  |  |  |  |  |  | ***C2*** | | c.841_849+19del, p.Val281_Arg283del | 5 | 0.0057 | NM_000063.6 | AR | |
| 8 | SIDS | 340 | 33 | 2 | Boy | 6 | Forensic | Yes | ***IKZF1*** | | c.52C>A, p.Pro18Thr | 3 | 8.2186e-06 | NM_006060.6 | AD | |
| 8 | SIDS |  |  |  |  |  |  |  | ***NLRP12*** | | c.1151C>T, p.Ala384Val | 3 | 8.21e-06 | NM_144687.4 | AD | |
| 8 | SIDS |  |  |  |  |  |  |  | ***SAMD9L*** | | c.1172A>G, p.Lys391Arg | 3 | 0 | NM_152703.5 | AD/som | |
| 8 | SIDS |  |  |  |  |  |  |  | ***SAMD9*** | | c.1304A>G, p.Lys435Arg | 3 | 0 | NM_017654.4 | AD/som | |
| 8 | SIDS |  |  |  |  |  |  |  | ***C8B*** | | c.73C>A(;)835A>G, p.Leu25Ile(;)Ile279Val | 3(;)3 | 0(;)7.44e-06 | NM_000066.4 | AR | |
| 8 | SIDS |  |  |  |  |  |  |  | ***WAS*** | | c.1013G>A, p.Arg338His | 3+ | 6.60e-06 | NM_000377.3 | XL | |
| 9 | SIDS | 246 | 40 | 4 | Girl | 19 | Forensic | Yes | ***RORC*** | | c.28C>T, p.Arg10* | 3 | 0.0027 | NM_005060.4 | AR | |
| 9 | SIDS |  |  |  |  |  |  |  | ***MBL2*** | | c.154C>T(;)161G>A, p.Arg52Cys(;)Gly54Asp | RF | 0.064(;)0.139 | NM_001378373.1 | AD | |
| 13 | SIDS | 189 | 36 | 2 | Boy | 11 | Forensic | Yes | ***SAMD9L*** | | c.3678dup, p.His1227Thrfs*15 | 3 | 1.24e-06 | NM_152703.5 | AD/som | |
| 13 | SIDS |  |  |  |  |  |  |  | ***MBL2*** | | c.161G>A, p.Gly54Asp | RF | 0.139 | NM_001378373.1 | AD | |
| 14 | SIDS | 182 | 40 | 4 | Girl | 9 | Forensic | Yes | ***STAT5B*** | | c.628T>C, p.Ser210Pro | 3 | 0 | NM_012448.4 | AD/AR | |
| 14 | SIDS |  |  |  |  |  |  |  | ***AIRE*** | | c.1249_1250insC, p.Leu417Profs*7 | 5 | 1.52e-05 | NM_000383.4 | AR (LOF) | |
| 14 | SIDS |  |  |  |  |  |  |  | ***MBL2*** | | c.161G>A, p.Gly54Asp | RF | 0.139 | NM_001378373.1 | AD | |
| 17 | SIDS | 295 | 40 | 3 | Girl | 77 | Forensic | Yes | ***CD247*** | | c.301C>T, p.Gln101* | 5 | 0.00027 | NM_198053.3 | AR | |
| 18 | SIDS | 154 | 40 | 4 | Boy | 33 | Forensic | Yes | ***TNFRSF1A*** | | c.805C>G, p.Pro269Ala | 3 | 0 | NM_001065.4 | AD | |
| 19 | SIDS | 224 | 39 | 3 | Boy | 24 | Forensic | Yes | ***TNFRSF13B (TACI)*** | | c.542C>A, p.Ala181Glu | 5 | 0.0054 | NM_012452.3 | AD/AR | |
| 20 | SIDS | 295 | 40 | 4 | Girl | 31 | Forensic | Yes | ***EOMES*** | | c.1966_1984del, p.Thr656Glyfs*16 | 3 | 0.00024 | NM_001278182.2 | U | |
| 20 | SIDS |  |  |  |  |  |  |  | ***C3*** | | c.2390A>G, p.Asp797Gly | 3 | 0 | NM_000064.4 | AR | |
| 21 | SIDS | 430 | 37 | 3 | Girl | 70 | Forensic | Yes | ***SAMD9*** | | c.4610A>G, p.Asn1537Ser | 3- | 6.16e-06 | NM_017654.4 | AD/som | |
| 27 | SIDS | 632 | 41 | 3 | Girl | 11 | Forensic | Yes | ***NFKBIA*** | | c.907C>G, p.Leu303Val | 3 | 1.32e-05 | NM_020529.3 | AD | |
| 28 | SIDS | 267 | 36 | 3 | Boy | 5 | Forensic | Yes | ***TREX1*** | | c.302A>C, p.Asp101Ala | 3 | 0 | NM_033629.6 | AD/AR | |
| 28 | SIDS |  |  |  |  |  |  |  | ***MBL2*** | | c.161G>A, p.Gly54Asp | RF | 0.139 | NM_001378373.1 | AD | |
| 29 | SIDS | 908 | 39 | 4 | Girl | 30 | Forensic | Yes | ***IRF2BP2*** | | c.707A>G, p.Lys236Arg | 3 | 3.32e-06 | NM_182972.3 | AD | |
| 29 | SIDS |  |  |  |  |  |  |  | ***SAMD9*** | | c.3437T>G, p.Val1136Gly | 3 | 0 | NM_017654.4 | AD/som | |
| 30 | SIDS | *na* |  |  | Girl | 41 | Forensic | Yes | ***IFIH1*** | | c.1804G>A, p.Glu602Lys | 3 | 0 | NM_022168.4 | AD | |
| 30 | SIDS |  |  |  |  |  |  |  | ***HOXA11*** | | c.193T>C, p.Tyr65His | 3 | 0 | NM_005523.6 | AD | |
| 35 | SIDS | 82 | 32 | 2 | Boy | 3 | Forensic | Yes | ***MBL2*** | | c.154C>T, p.Arg52Cys | RF | 0.064 | NM_001378373.1 | AD | |
| 36 | SIDS | 285 | 40 | 3 | Boy | 8 | Forensic | Yes | ***MBL2*** | | c.154C>T, p.Arg52Cys | RF | 0.064 | NM_001378373.1 | AD | |
| 38 | SIDS | 329 | 40 | 4 | Boy | 6 | Forensic | Yes | ***MBL2*** | | c.154C>T, p.Arg52Cys | RF | 0.064 | NM_001378373.1 | AD | |
| 40 | SIDS | 198 | 41 | 3 | Girl | 0 | Forensic | Yes | ***MBL2*** | | c.161G>A, p.Gly54Asp | RF | 0.139 | NM_001378373.1 | AD | |
| 41 | SIDS | 145 | 34 | 2 | Boy | 15 | Forensic | Yes | ***MBL2*** | | c.161G>A, p.Gly54Asp | RF | 0.139 | NM_001378373.1 | AD | |
| 42 | SIDS | 203 | 36 | 3 | Boy | 43 | Forensic | Yes | ***MBL2*** | | c.161G>A, p.Gly54Asp | RF | 0.139 | NM_001378373.1 | AD | |
| 43 | SIDS | 279 | 39 | 3 | Boy | 8 | Forensic | Yes | ***MBL2*** | | c.161G>A, p.Gly54Asp | RF | 0.139 | NM_001378373.1 | AD | |
| 44 | SIDS | 231 | 36 | 3 | Boy | 16 | Forensic | Yes | ***MBL2*** | | c.161G>A, p.Gly54Asp | RF | 0.139 | NM_001378373.1 | AD | |
| 45 | SIDS | *na* |  |  | Boy | 75 | Forensic | Yes | ***MBL2*** | | c.161G>A, p.Gly54Asp | RF | 0.139 | NM_001378373.1 | AD | |
| 49 | SIDS | *na* |  |  | Girl | 0 | Forensic | Yes | ***MBL2*** | | c.170G>A;(170G>A), p.Gly57Glu;(Gly57Glu) | 2 | 0.029 | NM_001378373.1 | AD | |
| 50 | SIDS | 250 | 38 | 3 | Boy | 21 | Forensic | Yes | ***MBL2*** | | c.170G>A, p.Gly57Glu | 2 | 0.029 | NM_001378373.1 | AD | |
| 51 | SIDS | 190 | 40 | 3 | Boy | 34 | Forensic | Yes | ***MBL2*** | | c.170G>A, p.Gly57Glu | 2 | 0.029 | NM_001378373.1 | AD | |
| 52 | SIDS | 191 | 29 | 2 | Boy | 3 | Forensic | Yes | ***MBL2*** | | c.170G>A, p.Gly57Glu | 2 | 0.029 | NM_001378373.1 | AD | |
| 53 | SIDS | 250 | 38 | 3 | Boy | 13 | Forensic | Yes | ***MBL2*** | | c.154C>T, p.Arg52Cys | RF | 0.064 | NM_001378373.1 | AD | |
| 54 | SIDS | 129 | 40 | 3 | Boy | 95 | Forensic | Yes | ***PLCG2*** | | c.[3532G>A], p.Ala1178Thr |  | 0 | NM_002661.5 | AD | |
| 55 | SIDS | 125 | 37 | 3 | Boy | 16 | Forensic | Yes | ***FCGR3B*** | | c.108C>G, p.Ser36Arg | 3 | 0.004 | NM_001244753.2 | U | |
| 55 | SIDS |  |  |  |  |  |  |  | ***ITPKB*** | | c.151G>A, p.Gly51Arg | 3 | 0.0001 | NM_002221.4 | AR | |
| 55 | SIDS |  |  |  |  |  |  |  | ***C6*** | | c.1352dupA, p.Tyr451* | 5 | 0.0001 | NM_000065.5 | AR | |
| 55 | SIDS |  |  |  |  |  |  |  | ***MASP2*** | | c.1921G>A, p.Asp641Asn | RF | 0.0002 | NM_006610.4 | AR | |
| 3 | Infection | 268 | 38 | 4 | Girl | 61 | Forensic | Yes | ***UNC13D*** | | c.2695C>T, p.Arg899* | 5 | 1.35e-05 | NM_199242.3 | AR | |
| 3 | Infection |  |  |  |  |  |  |  | ***MBL2*** | | c.161G>A, p.Gly54Asp | RF | 0.139 | NM_001378373.1 | AD | |
| 10 | Infection | 33 | 42 | 5 | Boy | 4 | *np* | *na* | ***CARMIL2*** | | c.245del, p.Pro82Leufs*127 | 5 | 0 | NM_001013838.3 | AR | |
| 10 | Infection |  | |  |  |  |  |  | ***MBL2*** | | c.161G>A, p.Gly54Asp | RF | 0.139 | NM_001378373.1 | AD | |
| 11 | Infection | 366 | 30 | 1 | Boy | 3 | *np* | Yes | ***SKIC3 (TTC37)*** | | c.3625C>T, p.Arg1209* | 5 | 4.34e-05 | NM_014639.4 | AR | |
| 12 | Infection | 22 | 30 | 2 | Boy | 0 | *np* | Yes | ***IRF2BP2*** | | c.616G>T, p.Gly206Cys | 3 | 2.31e-05 | NM_182972.3 | AD | |
| 15 | Infection | 216 | 41 | 4 | Girl | 9 | Medical | No | ***C8A*** | | c.1492C>T, p.Arg498* | 5 | 1.36e-05 | NM_000562.3 | AR | |
| 15 | Infection | |  |  |  |  |  |  | ***MBL2*** | | c.154C>T, p.Arg52Cys | RF | 0.064 | NM_001378373.1 | AD | |
| 16 | Infection | 42 | 38 | 2 | Boy | 18 | *np* | Yes | ***G6PD*** | | c.563C>T, p.Ser188Phe | 5 | 0.0014 | NM_001360016.2 | XL | |
| 16 | Infection |  | |  |  |  |  |  | ***FCN3*** | | c.70C>T, p.Gln24* | 5 | 8.05e-06 | NM_003665.4 | AR | |
| 16 | Infection |  | |  |  |  |  |  | ***DCLRE1C*** | | c.1903dup, p.Ser635Lysfs*6 | 3- | 0.00023 | NM_001033855.3 | AR | |
| 22 | Infection | 170 | 40 | 4 | Boy | 53 | Medical | *na* | ***MEFV*** | | c.2084A>G, p.Lys695Arg | 4 | 0.004 | NM_000243.3 | AD/AR | |
| 22 | Infection |  | |  |  |  |  |  | ***C9*** | | c.162C>A, p.Cys54* | 5 | 0.0012 | NM_001737.5 | AR | |
| 22 | Infection |  | |  |  |  |  |  | ***MBL2*** | | c.154C>T, p.Arg52Cys | RF | 0.064 | NM_001378373.1 | AD | |
| 23 | Infection | 172 | 31 | 2 | Boy | 2 | Forensic | *na* | ***MAGT1*** | | c.92C>T, p.Ala31Val | 3 | 3.97e-05 | NM_032121.5 | XL | |
| 23 | Infection |  | |  |  |  |  |  | ***ZAP70*** | | c.747C>A, p.Cys249* | 5 | 1.59e-06 | NM_001079.4 | AR | |
| 23 | Infection |  | |  |  |  |  |  | ***MBL2*** | | c.154C>T, p.Arg52Cys | RF | 0.064 | NM_001378373.1 | AD | |
| 24 | Infection | 160 | 39 | 3 | Girl | 71 | Forensic | No | ***MTHFD1*** | | c.1986delA, Phe664Leufs*2 | 3 | 2.18e-05 | NM_005956.4 | AR | |
| 24 | Infection |  | |  |  |  |  |  | ***GBA*** | | c.1226A>G, p.Asn409Ser | 5 | 0.001997 | NM_000157.4 | AR | |
| 25 | Infection | 28 | 35 | 2 | Girl | 79 | *np* | *na* | ***STAT5B*** | | c.616C>T, p.Gln206* | 5 | 0 | NM_012448.4 | AR (LOF) | |
| 25 | Infection |  | |  |  |  |  |  | ***CFI*** | | c.355G>A, p.Gly119Arg | 3+ | 0.00042 | NM_000204.5 | AR | |
| 25 | Infection |  | |  |  |  |  |  | ***MBL2*** | | c.154C>T(;)161G>A, p.Arg52Cys(;)Gly54Asp | RF | 0.064(;)0.139 | NM_001378373.1 | AD | |
| 26 | Infection | 275 | 36 | 3 | Boy | 15 | Forensic | *na* | ***VPS45*** | | c.55G>A, p.Gly19Arg | 3 | 1.59e-06 | NM_007259.5 | AR | |
| 31 | Infection | *np* | *na* | *na* | Girl | 68 | Forensic | No | ***TMC8*** | | c.1481del, p.Leu494Argfs*5 | 5 | 0.00025 | NM_152468.5 | AR | |
| 32 | Infection | 180 | 39 | 4 | Boy | 1 | Forensic | *na* | ***MBL2*** | | c.154C>T, p.Arg52Cys | RF | 0.064 | NM_001378373.1 | AD | |
| 33 | Infection | 125 | 31 | 2 | Boy | 2 | Medical | Yes | ***MBL2*** | | c.154C>T, p.Arg52Cys | RF | 0.064 | NM_001378373.1 | AD | |
| 34 | Infection | 106 | 30 | 2 | Boy | 5 | Forensic | *na* | ***MBL2*** | | c.154C>T, p.Arg52Cys | RF | 0.064 | NM_001378373.1 | AD | |
| 37 | Infection | 499 | 39 | 3 | Boy | 1 | Medical | Yes | ***MBL2*** | | c.154C>T, p.Arg52Cys | RF | 0.064 | NM_001378373.1 | AD | |
| 39 | Infection | 229 | 36 | 3 | Girl | 2 | *np* | Yes | ***MBL2*** | | c.154C>T, p.Arg52Cys | RF | 0.064 | NM_001378373.1 | AD | |
| 46 | Infection | 121 | 37 | 4 | Boy | 78 | Forensic | Yes | ***MBL2*** | | c.161G>A;(161G>A), p.Gly54Asp;(Gly54Asp) | RF | 0.139 | NM_001378373.1 | AD | |
| 47 | Infection | 517 | 41 | 3 | Boy | 4 | *np* | Yes | ***MBL2*** | | c.161G>A;(161G>A), p.Gly54Asp;(Gly54Asp) | RF | 0.139 | NM_001378373.1 | AD | |
| 48 | Infection | 192 | 29 | 1 | Boy | 7 | Forensic | *na* | ***MBL2*** | | c.161G>A;(161G>A), p.Gly54Asp;(Gly54Asp) | RF | 0.139 | NM_001378373.1 | AD | |
| **Pt #** | **Cause of death** | **T REC (µl)** | **GA (w)** | **Birth weight (kg)** | **Sex** | **Age (w)** | **Autopsy** | **Sudden death (<24h)** | **IEI-related variant** | **Significant findings** | | | | | |  |
| 56 | SIDS | 250 | 40 | 4 | Boy | 74 | *np* | Yes | No | CRP >100 mg/L, lung infiltrates | | | | | |  |
| 58 | SIDS | 326 | 33 | 2 | Boy | 3 | Forensic | Yes | No | none | | | | | |  |
| 59 | SIDS | 354 | 40 | 3 | Boy | 79 | Forensic | Yes | No | Enlarged spleen and liver, tonsils with pus, chronic inflammatory cells | | | | | |  |
| 60 | SIDS | 366 | 38 | 3 | Boy | 20 | Forensic | Yes | No | Nasopharyngeal sample positive for *Parainfluenza virus type 3* | | | | | |  |
| 61 | SIDS | 202 | 40 | 3 | Boy | 3 | Forensic | Yes | No | none | | | | | |  |
| 62 | SIDS | 640 | 39 | 3 | Boy | 7 | Forensic | Yes | No | none | | | | | |  |
| 63 | SIDS | 521 | 41 | 3 | Girl | 3 | Forensic | Yes | No | none | | | | | |  |
| 65 | SIDS | 341 | 33 | 2 | Girl | 3 | Forensic | Yes | No | Positive blood culture for *Staphylococcus aureus* | | | | | |  |
| 67 | SIDS | 308 | 40 | 3 | Boy | 60 | Forensic | Yes | No | none | | | | | |  |
| 68 | SIDS | 1060 | 40 | 4 | Girl | 15 | Forensic | Yes | No | none | | | | | |  |
| 69 | SIDS | 189 | 35 | 4 | Boy | 37 | Forensic | Yes | No | *na* | | | | | |  |
| 70 | SIDS | 494 | 37 | 3 | Boy | 95 | Forensic | Yes | No | Chronic bronchitis and mild respiratory infection, hospitalised twice due to infection prior to death (pneumonia and upper airway infection) | | | | | |  |
| 74 | SIDS | 163 | 37 | 3 | Boy | 9 | Forensic | Yes | No | none | | | | | |  |
| 75 | SIDS | 395 | 39 | 3 | Boy | 21 | Forensic | Yes | No | Multiorgan failure | | | | | |  |
| 76 | SIDS | 261 | 40 | 3 | Girl | 18 | Forensic | Yes | No | *na* | | | | | |  |
| 78 | SIDS | 159 | 41 | 5 | Boy | 10 | Forensic | Yes | No | none | | | | | |  |
| 79 | SIDS | *na* | *na* | *na* | Boy | 75 | Forensic | Yes | No | Extensive lung infiltrates | | | | | |  |
| 57 | Infection | 240 | 39 | 3 | Boy | 11 | *np* | No | No | CRP >100 mg/L, interstitial pneumonia, *Respiratory syncytial virus* | | | | | |  |
| 64 | Infection | 335 | 40 | 3 | Boy | 73 | Forensic | No | No | Sepsis, asplenia, pneumococcus | | | | | |  |
| 66 | Infection | 216 | 37 | 2 | Boy | 3 | Forensic | Yes | No | Cytomegalovirus | | | | | |  |
| 71 | Infection | 320 | 41 | 3 | Girl | 73 | Forensic | No | No | Group A Streptococcus, sepsis | | | | | |  |
| 72 | Infection | 499 | 40 | 4 | Boy | 1 | Medical | Yes | No | Sepsis | | | | | |  |
| 73 | Infection | 93 | 34 | 2 | Girl | 7 | Forensic | *na* | No | CRP>100 mg/L, positive blood culture for *Staphylococcus aureus* | | | | | |  |
| 77 | Infection | 409 | 39 | 3 | Boy | 7 | Forensic | *na* | No | *na* | | | | | |  |
| Abbreviations: Pt=patient; w=week; kg=kilogram; h=hour; TREC=T-cell receptor exicion circles; µl=microlitre**;** GA=gestational age; ACMG=The American College of Medical Genetics and Genomics' variant classification; GnomAD v4.0=genome aggregation database version 4.0; Inh=inheritance; IEI=inborn errors of immunity; SIDS=sudden infant death syndrome; AD=autosomal dominant; AR=autosomal recessive; LOF=loss-of-function variant; na=not available; RF=risk factor; VUS=variants of uncertain significance; XL=X-linked; 2=likely benign; 3=VUS; 4=likely pathogenic; 5=pathogenic; np=not performed; som=somatic; U=unknown. | | | | | | | | | | | | | | | | |
